# Supplementary material for: Unusual Dependence of the Diamond Growth Rate on the Methane Concentration in the Hot Filament Chemical Vapor Deposition Process
Source: Materials (Basel). 2021 Jan 16;14(2):426. doi: 10.3390/ma14020426 (PMC7830984; doi:10.3390/ma14020426)
Supplement: Supplementary file 1 [file materials-14-00426-s001.pdf]

materials

MDPI

Supplementary Materials

Unusual Dependence of the Diamond Growth Rate on the Me-Thane Concentration in the Hot Filament Chemical Vapor Deposition Process

Byeong-Kwan Song [1,†], Hwan-Young Kim [1,†], Kun-Su Kim [1], Jeong-Woo Yang [1] and Nong-Moon Hwang [1,2,*]

1 Department of Materials Science and Engineering, Seoul National University, 1 Gwanak-ro, Gwanak-gu, Seoul 08826, Korea; sbk32131832@snu.ac.kr (B.-K.S.); welcome777@snu.ac.kr (H.-Y.K.); lstatsl@snu.ac.kr (K.-S.K.); jwoo5432@snu.ac.kr (J.-W.Y.)
2 Research Institute of Advanced Materials, 599 Gwanak-ro, Gwanak-gu, Seoul 08826, Korea
* Correspondence: nmhwang@snu.ac.kr
† The authors contribute equally.

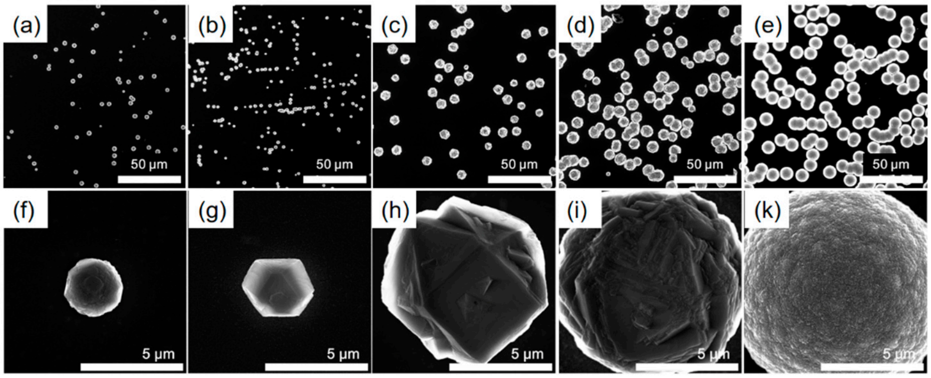

Figure S1. FESEM images of diamond particles deposited for 8 h on the Si substrate at a filament temperature of 2100 °C. The low magnification images are for (**a**) 0.3% CH_4 –99.7% H_2, (**b**) 0.5% CH_4 –99.5% H_2, (**c**) 1% CH_4 –99% H_2, (**d**) 1.5% CH_4 –98.5% H_2, and (**e**) 3% CH_4 –97% H_2. The high magnification images are for (**f**) 0.3% CH_4 –99.7% H_2, (g) 0.5% CH_4 –99.5% H_2, (**h**) 1% CH_4 –99% H_2, (**i**) 1.5% CH_4 –98.5% H_2, and (**k**) 3% CH_4 –97% H_2. The diamond particles were deposited before the overhaul of the chamber. The growth rate of diamond particles increased with increasing methane concentration from 0.3% to 3% at the filament temperature of 2100 °C.

Publisher's Note: MDPI stays neutral with regard to jurisdictional claims in published maps and institutional affiliations.

Copyright: © 2021 by the authors. Submitted for possible open access publication under the terms and conditions of the Creative Commons Attribution (CC BY) license (http://creativecommons.org/licenses/by/4.0/).

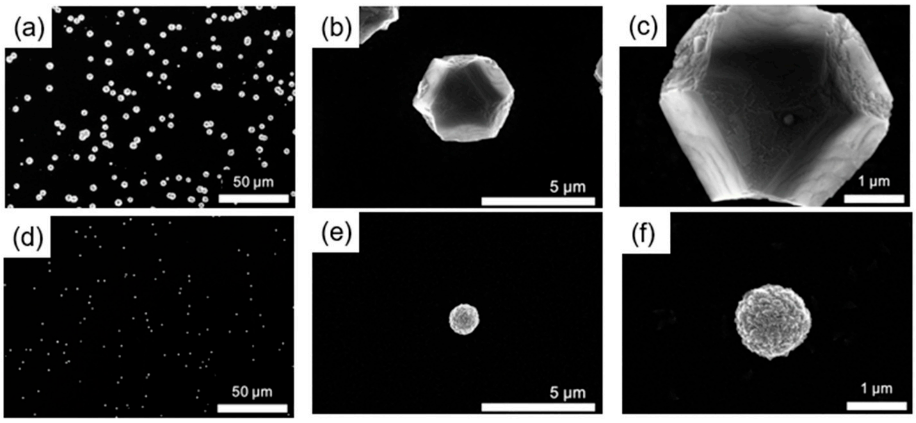

Figure S2. FESEM images of diamond particles deposited for 4 h on the Si substrate at a filament temperature of 1900 °C. FESEM images of diamond particles in (**a**), (**b**), and (**c**), which were deposited at 1% CH4 –99% H2, have different magnifications as indicated by the scale bar. FESEM images of diamond particles in (**d**), (**e**), and (**f**), which were deposited at 3% CH4 –97% H2, have different magnifications as indicated by the scale bar. The growth rate of diamond particles decreased with increasing the methane concentration from 1% to 3% at the filament temperature of 1900 °C.

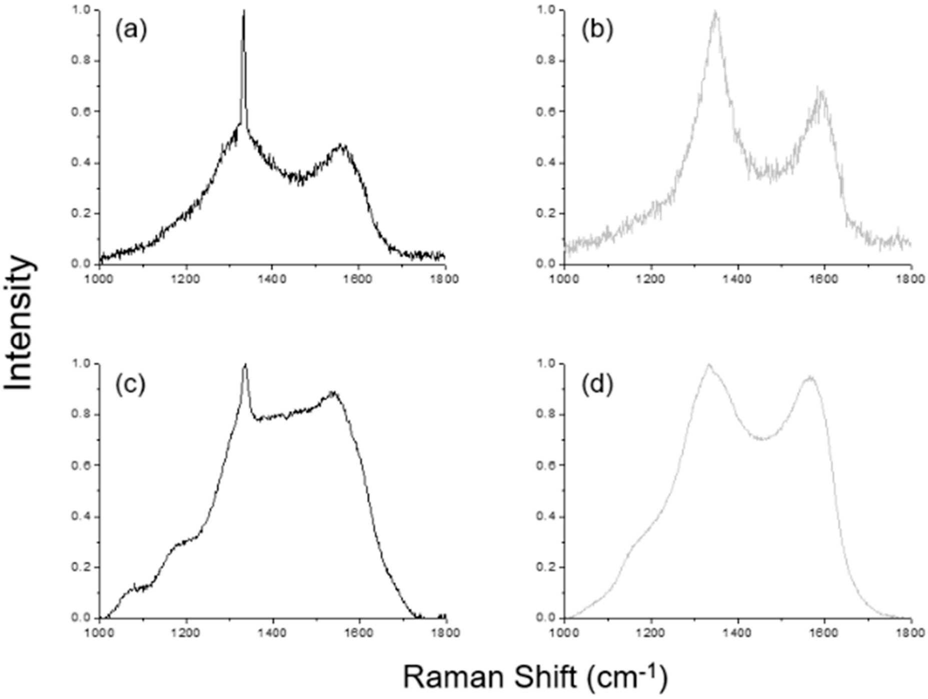

Figure S3. Raman spectra of deposited particles in Figures 2 and 3. (**a**) Raman spectra of Figure 2d, where the diamond particle was deposited at the filament temperature of 2100 °C and the gas mixture of 1% CH_4 –99% H_2. (**b**) Raman spectra of Figure 2h, where the diamond particle was deposited at the filament temperature of 2100 °C and the gas mixture of 3% CH_4 –97% H_2. (**c**) Raman spectra of Figure 3d, where the diamond particle was deposited at the filament temperature of 1900 °C and the gas mixture of 1% CH_4 –99% H_2. (**d**) Raman spectra of Figure 3h, where the diamond particle was deposited at the filament temperature of 1900 °C and the gas mixture of 3% CH_4 –97% H_2.
